# *EGFR*基因rs2293347多态对吉非替尼治疗非小细胞肺癌临床疗效的影响

**DOI:** 10.3779/j.issn.1009-3419.2011.08.02

**Published:** 2011-08-20

**Authors:** 飞 马, 兵河 徐, 东昕 林, 瞳 孙, 远凯 石

**Affiliations:** 1 100021 北京，中国医学科学院肿瘤医院肿瘤研究所内科 Department of Medical Oncology, Cancer Institute & Hospital, Chinese Academy of Medical Science, Peking Union Medical College, Beijing 100021, China; 2 100021 北京，中国医学科学院肿瘤医院病因及癌变研究室 Department of Etiology and Carcinogenesis, Cancer Institute & Hospital, Chinese Academy of Medical Science, Peking Union Medical College, Beijing 100021, China

**Keywords:** 肺肿瘤, EGFR, 吉非替尼, 单核苷酸多态性, Lung neoplasms, Epidermal growth factor receptor (EGFR), Gefitinib, Single nucleotide polymorphism

## Abstract

**背景与目的:**

表皮生长因子受体（epidermal growth factor receptor, *EGFR*）基因遗传变异可能影响蛋白的功能，从而影响EGFR抑制剂的疗效，本研究旨在探讨EGFR酪氨酸激酶抑制剂吉非替尼的临床疗效与靶标基因*EGFR*单核苷酸多态rs2293347之间的相关性。

**方法:**

对88例接受过吉非替尼治疗的晚期非小细胞肺癌（non-small cell lung cancer, NSCLC）患者进行分析，采用限制性片断长度多态进行基因分型，分析*EGFR*基因rs2293347多态与患者临床疗效及预后的关系。

**结果:**

*EGFR*基因rs2293347多态与吉非替尼的疗效相关，携带GG基因型的患者接受吉非替尼治疗的临床获益率为71.4%，而携带GA/AA基因型的患者仅为36.0%（*P*=0.002）。GG基因型的患者无进展生存期也明显长于GA/AA基因型的患者（10个月*vs* 3个月，*P*=0.005），但是两组的总生存时间（overall survival, OS）无统计学差异（*P*=0.409）。

**结论:**

*EGFR*基因rs2293347多态与吉非替尼临床疗效密切相关，可能成为预测吉非替尼疗效的理想的遗传标记物。

## Abstract (en)

**Background and objective:**

Genetic variations of the epidermal growth factor receptor (EGFR) may alter the protein function and therapeutic efficacy of EGFR inhibitors. The aim of this study is to investigate the association between single nucleotide polymorphism rs2293347 in EGFR and the clinical outcome in patients with advanced non-small cell lung cancer (NSCLC) treated with gefitinib.

**Methods:**

A total of 88 advanced NSCLC patients treated with gefitinib were analyzed in the present study. Polymerase chain reaction-restriction fragment length polymorphism (PCR-RFLP) method was conducted to analyze the genotype. The association study was performed between genotypes and clinical efficacy among 88 patients.

**Results:**

rs2293347 was associated with the efficacy of gefitinib. The response rate for the rs2293347 GG was significantly higher than that for the GA or AA (71.4% *vs* 36.0%, *P*=0.002). rs2293347 GG genotype was also associated with longer progression-free survival compared with GA or AA genotype (10 months *vs* 3 months, *P*=0.005). No significant difference was shown on the overall survival (OS) (*P*=0.409).

**Conclusion:**

rs2293347 polymorphism in exon 25 is associated with the clinical efficacy of gefitinib and may be a potential biomarker to predict the clinical outcome in advanced NSCLC patients treated with gefitinib.

吉非替尼（gefitinib, ZD1839）是近年来肿瘤靶向治疗成功的典范，属于EGFR受体酪氨酸激酶抑制剂，目前主要应用于晚期非小细胞肺癌（non-small cell lung cancer, NSCLC）的临床治疗^[1]^。吉非替尼从研发到临床应用以来，药物疗效相关因素的研究就一直是热门。研究^[2]^提示遗传相关因素可能是影响吉非替尼疗效的关键性因素。而单核苷酸多态性是最常见的遗传变异形式，也是导致许多药物疗效个体差异的重要因素^[3]^。本课题组曾通过药物靶标基因-表皮生长因子受体（epidermal growth factor receptor, *EGFR*）基因的全基因筛选策略，发现*EGFR*基因rs2293347多态可能与吉非替尼的疗效相关^[4]^，为此进行了深入的临床研究。

## 1 材料与方法

### 1.1 临床资料

用于单核苷酸多态性分析的病例均来自中国医学科学院肿瘤医院2004年1月-2006年12月接受吉非替尼单药治疗的晚期NSCLC患者，共88例。所有患者均经组织学或细胞学诊断，治疗前均具有可测量的靶病灶。其中男性39例，女性49例，中位年龄56岁（34岁-76岁）。患者PS评分均为0分-1分，80.5%的患者为非吸烟者，80.5%的患者为腺癌，其余19.5%为鳞癌或大细胞癌。所有患者均接受了吉非替尼的单药治疗，其中47例为二线治疗，41例为三线治疗。均采用RECIST标准评价近期疗效，1例患者达到完全缓解（complete response, CR），32例患者达到部分缓解（partial response, PR），21例患者接受吉非替尼治疗后病情稳定（stable disease, SD）超过24周，其余34例患者在治疗24周内出现病情进展（progressive disease, PD），总体有效率为37.5%，将CR、PR、SD定义为吉非替尼治疗有效（临床获益），PD患者定义为治疗无效，临床获益率为61.4%。

### 1.2 rs2293347基因分型

采用PCR-RFLP（限制性片断长度多态）的方法进行基因分型。分别采集所有患者抗凝外周静脉血2 mL，酚-氯仿法提取模板DNA。采用PCR的方法扩增*EGFR*基因第25外显子片段，PCR反应的引物分别为5’-ATGAGGTACTCGTCGGCATC-3’和5’-GAACCAAGGGGGATTTCATT-3’，产物大小为244 bp。25 μL PCR混合液中含0.1 μg模板DNA，引物浓度0.4 μmol/L，扩增条件为：95 ℃预变性2 min后，于94 ℃ 30 s、60 ℃ 30 s和72 ℃ 30 s进行40个循环，最后72 ℃延伸7 min。PCR产物采用限制性内切酶Tfi Ⅰ进行酶切，酶切温度60 ℃，酶切产物经凝胶电泳分析，读取基因型。

### 1.3 统计学分析

所有资料应用SPSS 12.0软件进行统计学分析，rs2293347基因型与临床特征及近期疗效关系的分析采用χ^2^检验，生存分析采用*Kaplan-Meier*方法，*P* < 0.05为差异有统计学意义。

## 2 结果

### 2.1 rs2293347基因分型结果

采用PCR-RFLP法对88例晚期NSCLC患者的外周血DNA进行了rs2293347基因分型。其中GG基因型63例，占71.6%；GA基因型24例，AA基因型1例，GA/AA基因型占28.4%。

### 2.2 rs2293347基因型与患者临床病理特征及吉非替尼近期疗效的关系

在88例接受吉非替尼单药治疗的晚期NSCLC患者中，将rs2293347不同基因型与患者临床病理特征以及吉非替尼的近期疗效进行分析，结果发现携带GG基因型的63例患者中，CR 1例（1.6%），PR 28例（44.4%），SD 16例（25.4%），PD 18例（28.6%），临床获益率为7 1. 4 %；携带A A基因型患者仅为1例，吉非替尼的临床疗效为PD；携带G A基因型患者为24例，其中PR 4例（16.7%），SD 5例（20.8%），PD 15例（62.5%），临床获益率仅为37.5%；携带GA/AA基因型患者临床获益率为36.0%。所有治疗有效的患者中，GG基因型占83.3%，GA/AA基因型占16.7%；所有治疗无效的患者中，GG基因型占52.9%，GA/A A基因型占47.1%。rs2293347基因型与吉非替尼的近期疗效密切相关（*P*=0.002），但是与患者的年龄、性别、PS评分、吸烟状况和病理类型均无明显相关性（表 1）。

**1:** 88例吉非替尼治疗患者临床特征与EGFR基因rs2293347基因型的统计分析 Stratification analysis of EGFR rs2293347 genotypes and clinical characteristics in 88 NSCLC patients treated with gefitinib

Characteristic	rs2293347 genotype		*P*
	GG [*n* (%)]	GA/AA [*n* (%)]	
Age (years)			0.469
< 60	35 (68.6)	16 (31.4)	
≥60	28 (75.7)	9 (24.3)	
Gender			0.661
Male	27 (69.2)	12 (30.8)	
Female	36 (73.5)	13 (26.5)	
WHO performance status			0.802
0	16 (69.6)	7 (30.4)	
1	47 (72.3)	18 (27.7)	
Smoking status			0.597
Non-smoker	49 (70.0)	21 (30.0)	
Smoker	13 (76.5)	4 (23.5)	
Histological subtype			0.677
Adenocarcinoma	50 (71.4)	20 (28.6)	
Other^a^	13 (76.5)	4 (23.5)	
Gefitinib therapy			0.759
2nd line	33 (70.2)	14 (29.8)	
3rd line	30 (73.2)	11 (26.8)	
Clinical response			0.002
Nonresponder^b^	18 (52.9)	16 (47.1)	
Responder^c^	45 (83.3)	9 (16.7)	
^a^Includes squamous cell carcinoma (*n* =12) and large cell carcinoma (*n* =5); ^b^Includes progressive disease; cIncludes complete response, partial response and stable disease.			

### 2.3 rs2293347不同基因型患者的预后

rs2293347 GG基因型NSCLC患者经吉非替尼治疗后，中位无进展生存期（progression free survival, PFS）为10个月，而rs2293347 GA/AA基因型患者的中位PFS为3个月，两组相比有统计学差异（*P*=0.005）。GG基因型患者的中位总生存期（overall survival, OS）也比GA/AA基因型患者长（22个月*vs* 14个月），但两组间差异无统计学意义（*P*=0.409）。

## 3 讨论

分子靶向治疗是近年肿瘤治疗学领域新的里程碑，特异性抑制肿瘤细胞是分子靶向治疗区别于传统化疗药物的关键性特征，也正是这一特征，使得靶向治疗适应人群的选择性远比传统化疗要严格。另外，目前靶向治疗药物十分昂贵，盲目地滥用靶向药物是对医疗资源的极大浪费，这也要求靶向治疗只能选择性地应用于少数最可能获益的患者人群。因此，如何预测分子靶向治疗的疗效以及选择最可能获益的目标人群，成为当前肿瘤靶向治疗领域亟待解决的关键性临床问题。

在吉非替尼的临床实践中，探寻疗效预测指标一直是研究热点。目前认为亚裔、女性、腺癌或无吸烟史的NSCLC患者往往对吉非替尼比较敏感^[5]^；EGFR的表达、酪氨酸激酶活性以及EGFR下游信号转导也可能影响了药物的敏感性^[6]^。另外，*EGFR*基因的突变与吉非替尼敏感性密切相关^[7]^。

以上药物疗效相关因素研究中，不同研究者得到的结论不尽一致，甚至有些研究结论互相矛盾^[1, 8-10]^，因此这些指标在临床应用于预测吉非替尼药物疗效前还需要更为大宗的临床研究加以证实。此外以上指标并不能全面解释吉非替尼疗效的个体差异。其它一些指标的检测例如突变，操作烦琐、费用昂贵、肿瘤组织来源困难，均限制了其临床广泛应用。因此，寻找简便、经济、准确性高的吉非替尼疗效预测指标，对于指导NSCLC合理的个体化分子靶向治疗具有重大的临床意义。

目前研究提示遗传变异主要为基因多态性可能是吉非替尼药物疗效差异的主要来源。本研究组前期曾开展了吉非替尼疗效相关的多态性研究^[11]^，主要集中在重复序列数目差异（VNTR）。对NSCLC患者*EGFR*基因第1内含子区CA双核苷酸单序列重复（CA-SSR）多态性进行了基因分型，结果发现携带短CA重复序列的患者吉非替尼的近期有效率明显高于携带长重复序列的患者，但是CA-SSR多态性并不能影响吉非替尼治疗后患者的无进展生存和总生存情况。

在人类基因组中数量庞大的单核苷酸多态（single nucleotide polymorphism, SNP）才是个体间遗传差异的主要来源。借助于高密度SNP图谱的绘制和高通量基因分型技术的发展^[12, 13]^，我们曾针对药物靶标开展了全基因功能性SNP筛查，结果发现*EGFR*基因SNP位点rs2293347的基因型与吉非替尼的临床疗效可能相关^[4]^。本研究进一步确定了这种相关性，并且发现rs2293347还与患者的无进展生存密切相关。rs2293347作为同义SNP，位于*EGFR*基因第25外显子这一功能活性区，但是并没有导致EGFR受体氨基酸的改变（D994D）。虽然许多同义SNP也可影响mRNA稳定性、转录或剪切，从而影响蛋白表达、结构或功能^[14-16]^。但是rs2293347是否通过影响EGFR受体功能而对吉非替尼疗效产生影响，确切的机制也有待于进一步的研究。

另外，SNP作为遗传标记物预测吉非替尼的疗效，在某些方面具有优于传统预测指标（*EGFR*基因突变）的优势。目前临床上能够获得基因突变检测标本的患者仅约20%（来自ISEL研究的数据）^[17]^，而所有患者均可以获得SNP检测的外周血标本，并且SNP检测流程相对简便、成本低廉。当然，rs2293347能否最终应用于临床，补充甚至替代现有疗效预测指标，还有待大宗的前瞻性临床研究证实。
